# Chemokines Up-Regulated in Epithelial Cells Control Senescence-Associated T Cell Accumulation in Salivary Glands of Aged and Sjögren’s Syndrome Model Mice

**DOI:** 10.3390/ijms22052302

**Published:** 2021-02-25

**Authors:** Mie Kurosawa, Yosuke Shikama, Masae Furukawa, Rieko Arakaki, Naozumi Ishimaru, Kenji Matsushita

**Affiliations:** 1 Department of Oral Disease Research, National Center for Geriatrics and Gerontology, 7-430 Morioka-cho, Obu 474-8511, Japan; mkurosawa@ncgg.go.jp (M.K.); masae@ncgg.go.jp (M.F.); kmatsu30@ncgg.go.jp (K.M.); 2 Department of Oral Molecular Pathology, Tokushima University Graduate School of Biomedical Sciences, 3-18-15 Kuramoto-cho, Tokushima 770-8504, Japan; arakaki.r@tokushima-u.ac.jp (R.A.); ishimaru.n@tokushima-u.ac.jp (N.I.)

**Keywords:** sialadenitis, xerostomia, cellular senescence, immunosenescence, chemokines

## Abstract

Immunosenescence is characterized by age-associated changes in immunological functions. Although age- and autoimmune-related sialadenitis cause dry mouth (xerostomia), the roles of immunosenescence and cellular senescence in the pathogenesis of sialadenitis remain unknown. We demonstrated that acquired immune cells rather than innate immune cells infiltrated the salivary glands (SG) of aged mice. An analysis of isolated epithelial cells from SG revealed that the expression levels of the chemokine CXCL13 were elevated in aged mice. Senescence-associated T cells (SA-Ts), which secrete large amounts of atypical pro-inflammatory cytokines, are involved in the pathogenesis of metabolic disorders and autoimmune diseases. The present results showed that SA-Ts and B cells, which express the CXCL13 receptor CXCR5, accumulated in the SG of aged mice, particularly females. CD4^+^ T cells derived from aged mice exhibited stronger in vitro migratory activity toward CXCL13 than those from young mice. In a mouse model of Sjögren’s syndrome (SS), SA-Ts also accumulated in SG, presumably via CXCL12-CXCR4 signaling. Collectively, the present results indicate that SA-Ts accumulate in SG, contribute to the pathogenesis of age- and SS-related sialadenitis by up-regulating chemokines in epithelial cells, and have potential as therapeutic targets for the treatment of xerostomia caused by these types of sialadenitis.

## 1. Introduction

The prevalence of dry mouth (xerostomia) is significantly higher in women than in men and increases with age due, in part, to salivary gland hypofunction [1,2,3,4]. Dry mouth is also a common symptom among patients with several systemic diseases, such as diabetes and Sjögren’s syndrome (SS) [5,6]. SS, which is more prevalent in women than in men (at a ratio of 9:1), is an autoimmune disorder that is characterized by the chronic dysfunction and destruction of exocrine glands, mainly the salivary and lacrimal glands due to lesions chronically infiltrated by lymphocytes, and the majority of SS patients are postmenopausal women [5,7]. Lymphocytic infiltration has also been reported in the salivary glands of healthy subjects with age [8]. These findings indicate that lymphocytic infiltration resulting in salivary gland destruction is involved in both age- and SS-related dry mouth.

Cellular senescence is characterized by the irreversible arrest of cell proliferation and markedly altered gene expression that is often associated with a unique signature, called the senescence-associated secretory phenotype (SASP) [9]. Accumulating evidence recently indicated a role for immune aging (immunosenescence) in increases in proinflammatory traits with age, including various chronic inflammatory disorders, such as diabetes and autoimmunity [10]. Significant changes have been reported in overall T cell populations with age. The proportion of naïve (CD44^low^ CD62L^high^) cells has been shown to markedly decrease among CD4^+^ T cells, with an age-dependent increase in effector memory T cells (TEM: CD44^high^ CD62L^low^) [11,12]. Among TEM cells, a unique population expressing programmed cell death 1 (PD-1) and CD153 (tumor-necrosis factor superfamily member 8) increase with age in mice, and are known as senescence-associated T cells (SA-Ts) [13]. Increases in SA-Ts are suggested to be involved in some immune aging phenotypes, such as an impaired acquired immune capacity, increases in proinflammatory traits, and an elevated risk of autoimmunity [14]. Although SA-Ts are involved in the pathogenesis of metabolic disorders [15] and the autoimmune disease systemic lupus erythematosus [16], their role in age- and autoimmune-related sialadenitis, leading to salivary gland hypofunction, remains unknown. 

In the present study, we demonstrated that populations of innate immune cells, such as macrophages and dendritic cells decreased, whereas that of acquired immune cells, namely, lymphocytes, increased in the salivary glands of aged mice. An analysis of gene expression profiles in the epithelial cells of salivary glands revealed that the expression levels of the chemokine CXCL13 in salivary gland epithelial cells were markedly higher in aged mice than in young mice. The accumulation of B cells and SA-Ts, which express the CXCL13 receptor CXCR5, was observed in the salivary glands of aged mice, and the population and number of SA-Ts in the salivary glands were both significantly higher in aged female mice than in aged male mice. TEM cells derived from aged mice exhibited stronger in vitro migratory activity toward CXCL13 than those from young mice. The localization of SA-Ts around the epithelial cells of the salivary glands of aged mice was confirmed by the immunohistological analysis. The accumulation of SA-Ts was also confirmed in the salivary glands of SS-like model mice, but was not accompanied by that of B cells, similar to aged mice. These results indicate that the accumulation of SA-Ts may be a common pathogenesis of age- and autoimmune-related sialadenitis.

## 2. Results

### 2.1. Immune Cell Proportions in Salivary Glands of Aged Mice

**Figure 1:**
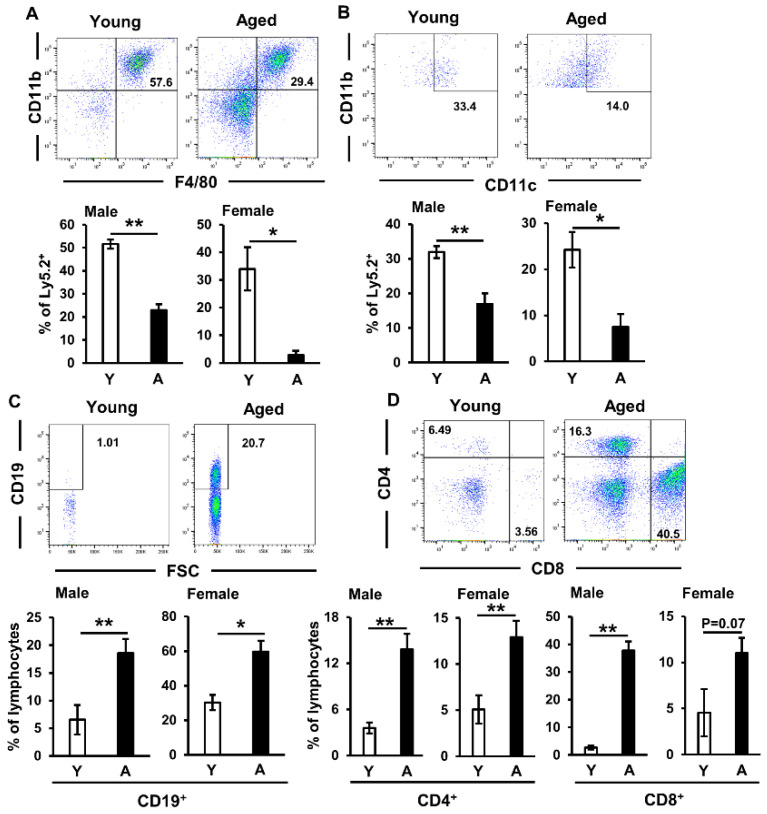
Lymphocyte accumulation in salivary glands of aged mice. (**A**,**B**) The frequencies of CD11b^+^ F4/80^+^ macrophages (**A**) and CD11b^+^ CD11c^+^ dendritic cells (**B**) gated on Ly5.2^+^ cells in the salivary glands of young (Y) and aged (A) C57BL/6N mice were assessed by flow cytometry. Left and right graphs show male and female mice, respectively (**A**: *n* = 4–5 mice per group, **B**: *n* = 3–5 mice per group). (**C**,**D**) The frequencies of CD19^+^ B cells (**C**) and CD4^+^ and CD8^+^ T cells (**D**) gated on lymphocytes in the salivary glands of young (Y) and aged (A) mice were assessed by flow cytometry. Left and right graphs show male and female mice, respectively (**C**: *n* = 4–6 mice per group, **D**: CD4 *n* = 4–6, CD8 *n* = 3–4 mice per group). Values are shown as means ± SEM. * *p* < 0.05 and ** *p* < 0.01 (the Student’s unpaired *t*-test).

Since innate [17,18] and acquired [5,19] immune cells are both involved in the pathogenesis of sialadenitis, we initially investigated the proportion of immune cells accumulating in the salivary glands of aged C57BL/6N mice. In the present study, submandibular glands were used as salivary glands. The infiltration of mononuclear cells, particularly around ductal cells, and the destruction of acinar cells were observed in the salivary glands of aged mice (Figure S1). A previous study reported hyposalivation in aged mice [20,21], and we also confirmed this in the aged mice used in the present experiments (Figure S2). Although no significant difference was observed between the cell numbers of macrophages (Figure S3A) and dendritic cells (Figure S3B) harvested from the salivary glands of young and aged mice, these cell proportions in the salivary glands were significantly lower in aged mice than in young mice (Figure 1A,B). However, a flow cytometric analysis revealed that the proportions of lymphocytes, namely, CD19^+^ B cells (Figure 1C) and CD4^+^ and CD8^+^ T cells (Figure 1D), were markedly elevated in aged mice. 

Moreover, the proportions of these cells, particularly CD19^+^ B cells (Figure 1C) and CD4^+^ T cells (Figure 1D), were significantly elevated in both male and female aged mice. These results indicated that salivary gland destruction by the infiltration of lymphocytes may be involved in age-related hyposalivation and sialadenitis.

### 2.2. Increases in CXCL13 Expression Levels in Salivary Gland Epithelial Cells of Aged Mice

**Figure 2:**
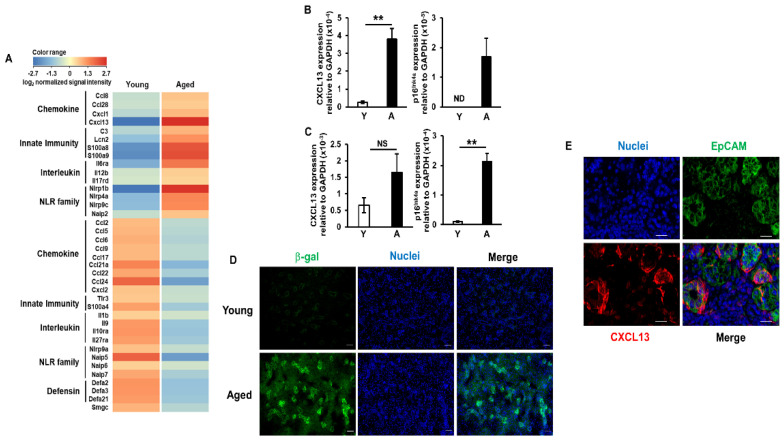
Salivary gland epithelial cells from aged mice display a unique gene expression phenotype. (**A**) Heatmap of gene expression levels of selected function-associated genes in isolated epithelial cells from young or aged C57BL/6N mice. Each column shows the expression level of a single sample. The log2-transformed ratio of normalized signal intensities versus the median signal intensity of these samples is shown. (**B**,**C**) CXCL13 and p16^Ink4a^ mRNA expression levels in salivary gland (**B**) or pulmonary (**C**) epithelial cells harvested from young (Y) or aged (A) mice, as analyzed by quantitative real-time PCR. Values are shown as means ± SEM (**B**: *n* = 6–9 mice per group, **C**: *n* = 5 mice per group). ND, not detected. ** *p* < 0.01. NS, not significant (the Student’s unpaired *t*-test). (**D**) Senescence-associated staining for SPiDER-βGal in frozen sections of salivary glands harvested from young and aged mice. Nuclei were stained with DAPI. Bars = 50 μm. (**E**) CXCL13 expression in the salivary glands of aged mice as detected by an immunofluorescence analysis using FITC-conjugated anti-mouse CD326 (EpCAM) mAb and an anti-mouse CXCL13 polyclonal antibody. Nuclei were stained with DAPI. Images show a Z-stack tile series of pictures. Bars = 20 μm.

Emerging evidence suggests that salivary gland epithelial cells are active participants in the pathogenesis of SS-related sialadenitis. For example, these cells express diverse signaling molecules involved in immune responses, including toll-like receptors (TLRs), major histocompatibility complex (MHC) classes I and II molecules, co-stimulators, chemokines, and cytokines [22]. Therefore, we investigated gene expression profiles in salivary gland epithelial cells isolated using magnetic cell sorting (MACS). Consistent with previous findings [23], the expression of EpCAM, an epithelial cell marker, was detected throughout the entire epithelia of the salivary glands of young and aged mice, particularly the ductal cells (Figure S4). A DNA microarray analysis revealed that the expression of 37 genes related to immune responses in salivary gland epithelial cells was markedly stronger or weaker in aged mice than in young mice (Figure 2A). Up-regulated genes in the salivary gland epithelial cells of aged mice mainly comprised chemokines, interleukins, and innate immune-related secretory proteins and receptors. Among these genes, we focused on CXCL13 (B-lymphocyte chemoattractant), which was the most strongly up-regulated gene (40-fold increase), and its receptor CXCR5, which is expressed by B cells and SA-Ts [16]. To assess expression profiles more quantitatively, we performed a real-time PCR analysis. CXCL13 mRNA expression levels in the salivary gland epithelial cells were significantly higher in aged mice (15-fold) than in young mice. Moreover, a marked increase in p16^INK4a^ mRNA expression levels, which has been reported in several tissues with age [20,24,25], was confirmed in cells harvested from aged mice (Figure 2B). Although p16^Ink4a^ mRNA expression levels were also significantly increased in pulmonary epithelial cells harvested from aged mice, those of CXCL13 were only slightly elevated by approximately 2.5-fold (Figure 2C). In assessments of senescence-associated beta-galactosidase (β-gal) activity using SPiDER-βGal staining [26], senescent cells were confirmed in the entire epithelia of the salivary glands of aged mice, particularly the ductal cells (Figure 2D). The results of an immunofluorescence analysis also confirmed that EpCAM-positive epithelial cells in the salivary glands of aged mice expressed CXCL13 (Figure 2E). These results suggest that elevated CXCL13 expression levels in salivary gland epithelial cells may contribute to the accumulation of lymphocytes, particularly B cells and SA-Ts, and subsequent tissue destruction. 

### 2.3. Involvement of Sex Differences in Lymphocyte Accumulation in Salivary Glands of Aged Mice

**Figure 3:**
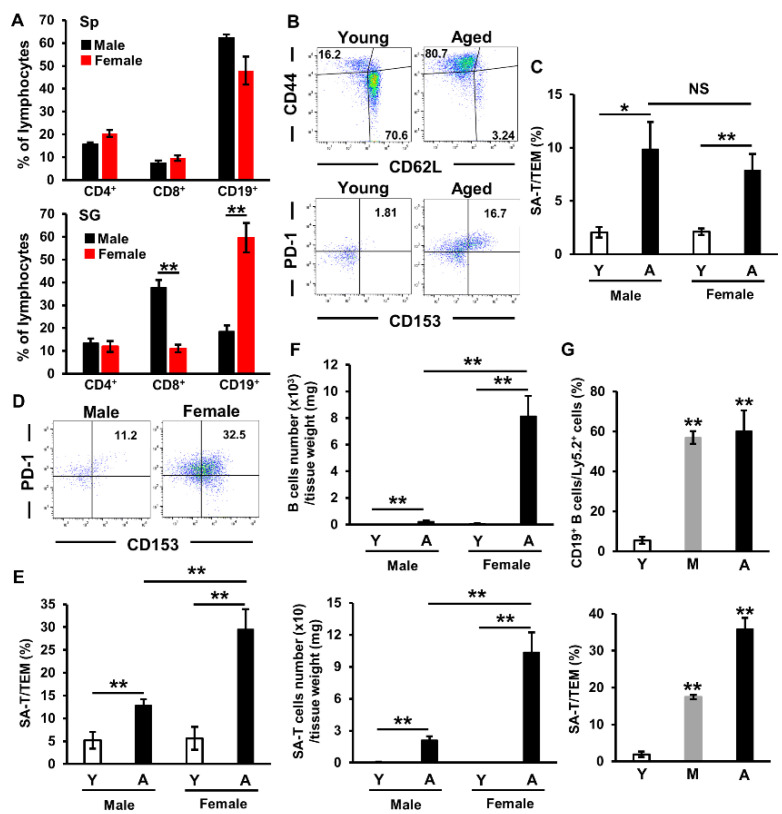
B cells and senescence-associated T cells (SA-Ts) accumulate in salivary glands of aged mice, particularly in female mice. (**A**) Proportion of lymphocytes in the spleen (Sp) and salivary glands (SG) of aged C57BL/6N mice (CD4^+^: *n* = 6–7 mice per group, CD8^+^: *n* = 4–5 mice per group, CD19^+^: *n* = 4–6 mice per group). (**B**) CD44/CD62L expression in CD4^+^ T cells and PD-1/CD153 expression in TEM cells in the spleens of young and aged mice as detected by a flow cytometric analysis. Results are representative of those from each group of mice. (**C**) Proportions of SA-Ts in the spleens of young (Y) and aged (A) mice (*n* = 5–6 mice per group). (**D**) PD-1/CD153 expression in TEM cells in the salivary glands of male and female aged mice as detected by a flow cytometric analysis. Results are representative of those from each group of mice. (**E**) Proportions of SA-Ts in the salivary glands of young (Y) and aged (A) mice (*n* = 5–6 mice per group). (**F**) Numbers of CD19^+^ B cells and SA-Ts in the salivary glands of young (Y) and aged (A) mice (*n* = 4–6 mice per group). (**G**) Proportions of CD19^+^ B cells and SA-Ts in the salivary glands of female young (Y), middle-aged (M), and aged (A) mice (*n* = 4–5 mice per group). Values are shown as means ± SEM. (**A**,**C**,**E**,**F**) * *p* < 0.05, ** *p* < 0.01, NS, not significant (the Student’s unpaired *t*-test). (**G**) ** *p* < 0.01 versus young mice (Dunnett’s multiple-comparison test).

Dry mouth is more prevalent in women, and sex has been shown to influence multiple aspects of acquired immunity [27]. Although no significant differences were observed in the proportion of splenic lymphocytes between male and female aged mice, we confirmed a sex difference in that of infiltrated lymphocytes in salivary glands, namely, the proportions of CD8^+^ T cells and CD19^+^ B cells were significantly smaller and larger in female aged mice than in male aged mice, respectively (Figure 3A). The proportion of TEM (CD44^high^ CD62L^low^) cells in the spleen was larger in aged mice than in young mice (Figure 3B), while that of SA-Ts (PD-1^+^CD153^+^) was also significantly larger; however, no significant differences were noted between male and female aged mice (Figure 3C). In the salivary glands, the proportion of SA-Ts was 2-fold larger in female aged mice than in male aged mice (Figure 3D,E). The numbers of B cells and SA-Ts in salivary glands were significantly higher (30- and 5-fold, respectively) in female aged mice than in male aged mice (Figure 3F). Moreover, the proportion of accumulated B cells peaked in middle-aged mice, whereas that of SA-Ts showed age-dependent increases in female mice (Figure 3G). These results indicate that the systemic conditions of females may be more inducible to the accumulation of B cells and SA-Ts, and that B cells accumulate in salivary glands before SA-Ts with age. 

### 2.4. Enhanced Migratory Response of Aged CD4^+^ T Cells to CXCL13

**Figure 4:**
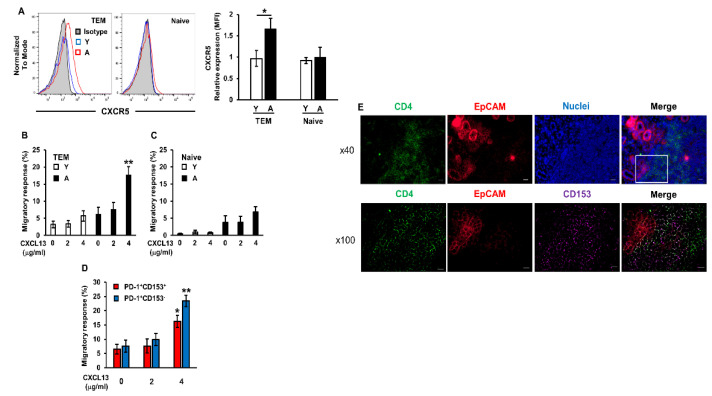
Enhanced migratory rate of CD4^+^ T cells toward CXCL13 isolated from aged mice. (**A**) The cell surface expression of CXCR5 on naïve and TEM cells from the spleens of young (Y) and aged (A) female C57BL/6N mice was detected by flow cytometry (*n* = 5–6 mice per group). Left histograms show a representative of those from each group of mice. Values are shown as means ± SEM. * *p* < 0.05 (the Student’s unpaired *t*-test). (**B**–**D**) Dose-dependent migratory response of TEM cells (**B**), naïve cells (**C**), and PD-1^+^CD153^+^ and PD-1^+^CD153^−^ TEM cells (**D**) to increasing concentrations of CXCL13 in aged female mice. Values are shown as the means ± SEM of tetraplicate measurements. These results are two independent experiments. * *p* < 0.05 and ** *p* < 0.01 versus the control group (the CXCL13 concentration is 0 μg/mL) (Dunnett’s multiple comparison test). (**E**) CD153^+^ CD4^+^ T cell localization in the SG of aged female mice was detected by an immunofluorescence analysis. Lower images show high magnification (×100) of the square area of the upper image. CD4 (green), EpCAM (red), CD153 (purple), CD4 and CD153 (white), and nuclei (blue). The scale bars of upper and lower images are 20 and 10 μm, respectively.

To examine whether the facilitation of migratory activity contributes to TEM cell infiltration in aged mice, the migratory response of CD4^+^ T cells isolated from young and aged mouse spleens to CXCL13 was assessed using an in vitro trans-well migration assay. The expression levels of the CXCL13 receptor CXCR5 were significantly higher in TEM cells isolated from aged mice than from young mice; however, its expression was not detected in naïve cells (Figure 4A). The migratory response to CXCL13 was significantly increased in TEM cells isolated from aged mice, but not in cells isolated from young mice (Figure 4B). In contrast, a migratory response toward CXCL13 was not observed in CD4^+^ naïve T cells isolated from young and aged mouse spleens (Figure 4C). There are two populations of TEM cells: SA-Ts (PD-1^+^ CD153^+^ CXCR5^low^) and T follicular helper (T_FH_) cells (PD-1^+^ CD153^−^ CXCR5^high^) which develop in association with germinal centers during antigen-driven immune responses. T_FH_ cells show no evidence of cellular senescence or CD153 expression and, thus, are distinct from SA-Ts [28]. We confirmed that the migratory response to CXCL13 was significantly increased in both PD-1^+^ CD153^+^ and PD-1^+^ CD153^−^ CD4^+^ T cells isolated from aged mice (Figure 4D). Moreover, the results of the immunohistological analysis confirmed that CD153^+^ CD4^+^ T cells localized around the EpCAM^+^ epithelial cells of the salivary glands of aged mice (Figure 4E). These results indicate that the up-regulated expression of CXCL13 in the salivary gland epithelial cells of aged mice enhance the migration of SA-Ts to these tissues. 

### 2.5. Accumulation of SA-Ts in Salivary Glands of SS Model Mice

**Figure 5:**
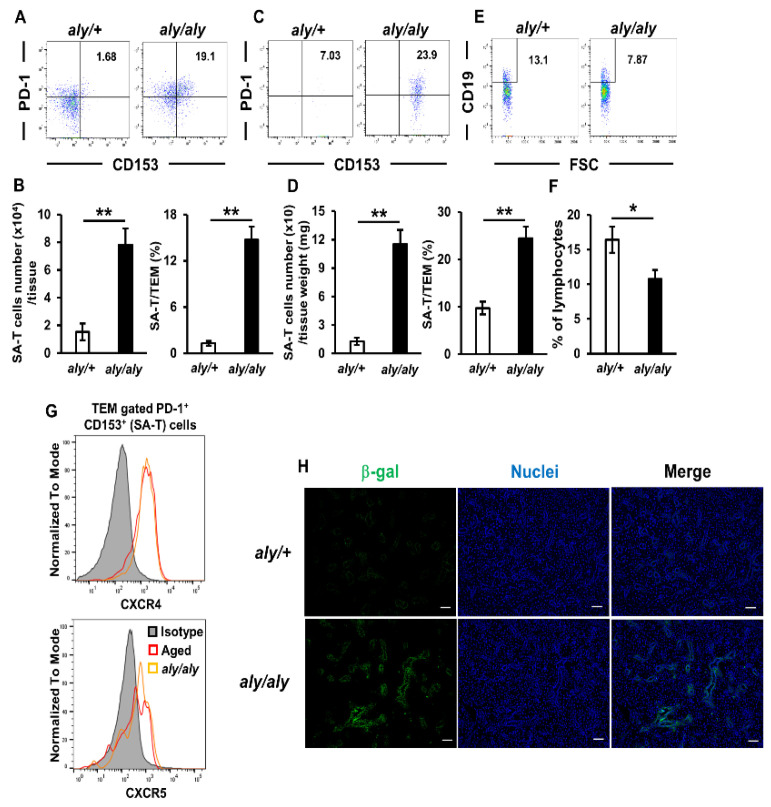
Increases in SA-Ts, but not B cells, in salivary glands of *aly/aly* mice. (**A**) PD-1^+^CD153^+^ expression in TEM cells in the spleens of *aly/+* and *aly/aly* female mice (age 16 weeks) as detected by a flow cytometric analysis. Results are representative of those from each group of mice. (**B**) Numbers and proportions of SA-Ts in the spleens of *aly/+* and *aly/aly* female mice (*n* = 4–5 mice per group). (**C**) PD-1^+^CD153^+^ expression in TEM cells in the salivary glands of *aly/+* and *aly/aly* female mice (age 16 weeks) as detected by a flow cytometric analysis. Results are representative of those from each group of mice. (**D**) Numbers and proportions of SA-Ts in the salivary glands of *aly/+* and *aly/aly* female mice (*n* = 3–4 mice per group). (**E**) The frequencies of CD19^+^ B cells gated on lymphocytes in the salivary glands of *aly/+* and *aly/aly* female mice (age 16 weeks) were assessed by flow cytometry. Results are representative of those from each group of mice. (**F**) Proportions of CD19^+^ B cells in the SG of *aly/+* and *aly/aly* female mice (age 16 weeks) (*n* = 3–5 mice per group). (**G**) The cell surface expression of CXCR4 and CXCR5 on SA-Ts from the spleens of aged and *aly/aly* female mice was detected by flow cytometry. Histograms show a representative of two independent experiments. Values are shown as means ± SEM. * *p* < 0.05 and ** *p* < 0.01 (the Student’s unpaired *t*-test). (**H**) Senescence-associated staining for SPiDER-βGal in frozen sections of salivary glands harvested from *aly/+* and *aly/aly* female mice (age 12 weeks). Nuclei were stained with DAPI. Bars = 50 μm.

Accumulating evidence indicates that SA-Ts are involved in the pathogenesis of many diseases, such as an autoimmune disease [16] and metabolic disorders [15]. Therefore, we investigated whether SA-Ts are present in the salivary glands of SS-like model (*aly/aly*) mice. In these mice, mononuclear cells mainly consisted of CD4^+^ cells that infiltrated the periducts of salivary glands, germinal center formation was not observed in the spleen, and the systemic absence of lymph nodes and Peyer’s patches was detected [19,29]. Although the generation and development of SA-Ts were previously shown to require germinal center formation and B cells [16], we confirmed that the number and proportion of SA-Ts in the spleen were significantly higher in *aly/aly* mice than in *aly/+* mice (Figure 5A,B). No significant differences were observed in the proportions of macrophages and dendritic cells in salivary glands between *aly/+* and *aly/aly* mice (Figure S5). Moreover, the number and proportion of SA-Ts in the salivary glands were also significantly higher in *aly/aly* mice than in *aly/+* mice (Figure 5C,D). However, the proportion of CD19^+^ B cells in the salivary glands was significantly lower in *aly/aly* mice than in *aly/+* mice (Figure 5E,F), which was a different pattern from that of aged mice (Figure 1C). Since the up-regulated expression of CXCL12 in the epithelial cells of target tissues has been shown to contribute to the greater accumulation of TEM cells in this mouse model [19], we investigated the cell surface expression of the CXCL12 receptor CXCR4 in SA-Ts. Similar to CXCR5, we confirmed the expression of CXCR4 in SA-Ts harvested from the spleens of both aged and *aly/aly* mice (Figure 5G). Senescence-associated β-gal activity in the ductal epithelial cells of the target tissue, which showed the up-regulated expression of CXCL12 [19], was stronger in *aly/aly* mice than in *aly/+* mice (Figure 5H). These results suggested that SA-Ts are also involved in the pathogenesis of SS-related sialadenitis and that CXCL12/CXCR4 signaling plays a role in the accumulation of SA-Ts toward target tissues. 

## 3. Discussion

To the best of our knowledge, this is the first study to show that the up-regulated expression of chemokines in epithelial cells of the salivary glands of aged and SS-like model mice is a critical factor promoting B-cell and/or SA-T accumulation and subsequent tissue destruction. SA-Ts exhibit the characteristic features of cellular senescence, with defective T cell receptor-mediated proliferation and T cell cytokine production. However, upon T cell receptor stimulation, SA-Ts secrete large amounts of atypical pro-inflammatory cytokines and chemokines, such as osteopontin, interferon (IFN)-γ, and CCL3 [16]. Osteopontin produced by SA-Ts was found to protect germinal center B cells from antigen-induced apoptosis [16]. Osteopontin transgenic mice, particularly females, exhibit the lymphocytic infiltration of salivary glands and hyposalivation, similar to SS [30]. Moreover, IFN-γ [31] and CCL3 [22] have been identified as active participants in the pathogenesis of SS. However, the molecular mechanisms by which SA-Ts are involved in age- and SS-related sialadenitis and hyposalivation have not yet been elucidated. Although the accumulation of SA-Ts was demonstrated in the present study, further studies are needed on the underlying mechanisms using murine and human samples. 

Aging-related changes have also been reported in cytotoxic T cells and regulatory T cells (Tregs) that act in concert to provide T cell-mediated immunity [32,33]. Tregs have functions that suppress immune responses activated by conventional T cells [34]. Previous studies suggested that aged Tregs were less effective at suppressing the function of conventional T cells [35] and expanding in response to muscle injury [36], implying that aging negatively influences the intrinsic function of Tregs. The number of peripheral Tregs was shown to be significantly lower in SS patients than in healthy controls [37]. Moreover, the frequency of Foxp3^+^ Tregs in salivary gland tissues from SS patients correlated with the inflammation grade and some risk factors for the development of lymphoma [38]. In the present study, we demonstrated that SA-Ts accumulated in the salivary glands of aged and SS-like model mice. In the future, we intend to investigate the involvement of aged Tregs in the pathogenesis of age- and SS-related sialadenitis.

Chemokines, which are small chemoattractant proteins that guide cellular migration, are strongly involved in the development of sialadenitis. The expression of CXCL13 in salivary tissue has been shown to increase with disease progression, while its blockage resulted in a modest reduction in glandular inflammation in SS model mice. Moreover, CXCL13 concentrations in the serum and saliva were significantly higher in patients with SS than in healthy controls [39]. In the present study, we demonstrated that CXCL13 was the most strongly up-regulated chemokine in the salivary gland epithelial cells of aged mice (Figure 2A). Although the mechanisms underlying the up-regulation of CXCL13 have not yet been elucidated in detail, Dragin et al. reported that a physiological concentration of estradiol induced a significant decrease in CXCL13 protein expression levels in thymic epithelial cells, and that CXCL13 mRNA expression levels were significantly higher in the thymus of aromatase (the enzyme involved in estrogen synthesis) knockout mice than in wild-type mice, suggesting that CXCL13 expression levels are reduced by estrogens [40]. Moreover, Liu et al. recently reported that CXCL13 mRNA expression levels were increased in murine macrophages with p16^INK4a^ promoter activation [41]. Based on the significantly higher numbers of B cells and SA-T cells, which express the CXCL13 receptor CXCR5, in the salivary glands of female aged mice than of male aged mice (Figure 3F), these results indicate that decreases in estrogen signaling and p16^INK4a^ activation exert additive or synergistic effects on CXCL13 expression in salivary gland epithelial cells. 

Dry mouth treatments, such as sialagogues and moisturizing agents, are mainly symptomatic, and a radical therapy has not yet been established [42]. The therapeutic targeting of senescence to promote healthy aging and prevent age-related diseases, otherwise known as senotherapy, has recently been attracting increasing attention [43]. Although SA-Ts are involved in the pathogenesis of metabolic disorders [15] and the autoimmune disease systemic lupus erythematosus [16], Yoshida et al. recently reported that the elimination of SA-T cells using a CD153 vaccine improved glucose tolerance and insulin sensitivity [44]. In the present study, we confirmed the accumulation of SA-Ts in the salivary glands of both aged and *aly/aly* mice, suggesting its involvement in the common pathogenesis of age- and SS-related sialadenitis. Although further studies are needed to elucidate the mechanisms underlying the overexpression of CXCL13 and CXCL12 in epithelial cells, the present results indicate that the CD153 vaccine is also effective for preventing age- and SS-related sialadenitis. Oxidative stress has also been implicated as one of the causes of aging and age-related diseases, such as type 2 diabetes [45,46]. Previous studies reported that local oxidative stress is involved in hyposalivation [47], and that astaxanthin, a carotenoid with antioxidant properties, attenuated age-related hyposalivation [48] in mice. Oxidative stress disrupts cell structures, and this function may be involved in the pathogenesis of salivary glands in SS [49,50]. These findings indicate that oxidative stress is also a target for the prevention of age- and SS-related hyposalivation.

We recently demonstrated that SA-Ts accumulated in the lacrimal glands of aged mice, but increased pilocarpine-stimulated tear secretion in aged mice, presumably via up-regulated peroxisome proliferator activated receptor-γ (PPARγ) and adiponectin-mediated signaling in the lacrimal glands of aged mice [51]. However, the DNA microarray analysis in this study revealed no marked differences in PPARγ mRNA expression levels in salivary gland epithelial cells isolated from young and aged mice, and no significant changes in the expression levels of adiponectin and its receptor AdipoR2 mRNA in the salivary glands of young and aged mice (Figure S6). PPARγ, which controls the secretion of adipokines, such as adiponectin [52], and its ligands exert not only therapeutic effects to increase tear fluid production [53], but also anti-inflammatory and anti-apoptotic effects in human salivary gland epithelial cells [54] and the amelioration of histopathological lesions in the salivary glands of SS model mice [55]. Although pioglitazone, a PPARγ ligand, is already on the market and its side effects are well known, these results indicate that it may be an attractive candidate due to its safety and effectiveness against age- and SS-related sialadenitis.

In conclusion, the present results demonstrated that lymphocytes rather than innate immune cells, such as macrophages and dendritic cells, accumulated in the salivary glands of aged mice. SA-Ts accumulated in the salivary glands of aged and *aly/aly* mice, presumably via the up-regulated expression of chemokines in these epithelial cells. The accumulation of B cells and SA-Ts occurred to a greater extent in female than in male aged mice. The present results will promote research with a focus on senescent cells as a valuable strategy for the prevention and treatment of age-and SS-related sialadenitis.

## 4. Materials and Methods

### 4.1. Animals

All animal experiments were approved by and conducted in accordance with guidelines established by the National Center for Geriatrics and Gerontology Animal Ethics Committee (29-4, 9/3/2017). Young adult C57BL/6N mice (age: 8–10 weeks), middle-aged adult C57BL/6N mice (age: 12 months), and aged adult C57BL/6N mice (age: 22–25 months) were obtained from Japan SLC Inc. (young and middle-aged: Hamamatsu, Japan) or the Experimental Animal Facility at the National Center for Geriatrics and Gerontology (aged: Obu, Japan). Alymphoplasia (*aly*)*/aly* and *aly/+* mice were obtained from CLEA Japan (Tokyo, Japan). Mice were housed in specific pathogen-free conditions under a 12-h light-dark photocycle and had *ad libitum* access to water and food. The temperature in the room was maintained at 23 ± 2 °C and humidity at 50 ± 10%.

### 4.2. Hematoxylin and Eosin Staining

Salivary glands were harvested from mice and fixed in 10% phosphate-buffered formaldehyde (pH 7.2). A histological examination was performed using sections stained with hematoxylin and eosin (MUTO PURE CHEMICALS CO., LTD., Tokyo, Japan).

### 4.3. Measurement of Saliva Secretion

Mice were anesthetized and intraperitoneally injected with 1 mg/kg of pilocarpine (Kanto Chemical Co., Inc., Tokyo, Japan). Saliva was collected from the mouth for 5 min using ring caps (AS ONE Corporation, Osaka, Japan). The total volume of saliva was measured and calculated per body weight.

### 4.4. RNA Isolation

Total RNA was extracted from cells and tissues using the RNeasy mini kit or RNeasy Lipid Tissue Mini Kit (Qiagen, Hilden, Germany), respectively, according to the manufacturer’s instructions. Total RNA concentrations were measured using a Nanodrop spectrophotometer (Thermo Fisher Scientific, Waltham, MA, USA), and cDNA was synthesized with the PrimeScript RT Master Mix (Takara Bio Inc., Shiga, Japan). In the DNA microarray, RNA quality was assessed using an Agilent 2100 bioanalyzer (Agilent Technologies, Santa Clara, CA, USA).

### 4.5. Quantitative Real-Time PCR Analysis 

PCR was performed on a LightCycler 96 system using FastStart Essential DNA Green Master (Roche Applied Science, Mannheim, Germany). The following primers were used for the amplification of specific genes: adiponectin, 5′- CAGGCATCCCAGGACATCC-3′ (sense) and 5′- CCAAGAAGACCTGCATCTCCTTT-3′ (antisense); AdipoR2, 5′-TTCCTATTATGAAAATAGCCCGGA-3′ (sense) and 5′-CATGATGGGAATGTAGGAGC-3′ (antisense); CXCL13, 5′-TGGCTGCCCCAAAACTGA-3′ (sense) and 5′-TGGCACGAGGATTCACACAT-3′ (antisense); p16^INK4a^, 5′-CGTACCCCGATTCAGGTGAT-3′ (sense) and 5′-TTGAGCAGAAGAGCTGCTACGT-3′ (antisense); GAPDH, 5′-GCCTTCCGTGTTCCTACCC-3′ (sense) and 5′-TGAAGTCGCAGGAGACAACC-3′ (antisense). The relative mRNA expression of each transcript was normalized against GAPDH mRNA.

### 4.6. DNA Microarray Analysis

Gene expression profiles were analyzed by Hokkaido System Science (Hokkaido, Japan) using the SurePrint G3 Mouse GE Microarray kit 8 × 60K (Agilent Technologies). Data acquisition and the normalization of expression data were performed using GeneSpring 14.9 (Agilent Technologies), and per-chip normalization to the 75th percentile was conducted. After this normalization, extremely low intensity probes were excluded, and differentially expressed genes were identified by applying a cut-off of ±2-fold. Heat maps displayed values normalized to baseline transformation to the median of all samples. Microarray data are available from the Gene Expression Omnibus database (https://www.ncbi.nlm.nih.gov/geo/, accessed on 20 December 2020) under accession number GSE156682.

### 4.7. Immune Cell Isolation from the Spleen and Salivary Glands

Spleen cells were suspended by homogenization, and red blood cells were removed with 0.83% ammonium chloride. The remaining cells were washed twice with 2% fetal bovine serum (FBS)–Dulbecco’s modified Eagle’s medium (DMEM: Sigma-Aldrich, St. Louis, MO, USA). Whole salivary gland lobes were minced into pieces of 1–3 mm, homogenized with a glass tissue homogenizer, and digested with 1 mg/mL collagenase type I (FUJIFILM Wako Pure Chemical Corporation, Osaka, Japan) in DMEM containing 10% FBS at 37 °C for 10 min. They were filtered through a 70-μm nylon mesh, centrifuged (600× *g*, 4 °C, 10 min), and rinsed twice with DMEM containing 2% (spleen) or 10% (salivary glands) FBS. The viability of isolated cells was assessed by a cell counter (Countess II Automated Cell Counters, Thermo Fisher Scientific) with trypan blue staining. Cell numbers were counted as the total absolute number of isolated cells per tissue by a cell counter (Countess II Automated Cell Counters) with trypan blue staining. The proportion of suspended cells was then analyzed by flow cytometry as described below. 

### 4.8. Isolation of Epithelial Cells from Salivary Glands and Lungs Using Magnetic Cell Sorting (MACS)

Tissues were minced and homogenized as described above and digested with 1 mg/mL collagenase type I (Wako), 1 mg/mL hyaluronidase type I (Sigma), and 0.01 mg/mL DNase I (Roche) in DMEM containing 10% FBS at 37 °C for 40 min. They were then filtered through a 70-μm nylon mesh, centrifuged (600× *g*, 4 °C, 5 min), rinsed twice with DMEM containing 10% FBS, and filtered through a 40-μm nylon mesh. Epithelial cells from the cell suspension were collected by positive selection using Miltenyi mouse CD326 (epithelial cell adhesion molecule; EpCAM) MicroBeads (130-105-958) [56]. We confirmed that purity was more than 80% (Figure S7). Total RNA was extracted as described above.

### 4.9. Flow Cytometric Analysis

Immune cells and epithelial cells harvested from tissues were stained using a PE-Cy7-conjugated anti-mouse CD4 monoclonal antibody (mAb) (100405), PE-Cy5-conjugated anti-mouse CD8a mAb (100709), PE-Cy5-conjugated anti-mouse/human CD11b mAb (101209), allophycocyanin (APC)-Cy7-conjugated anti-mouse CD11c mAb (17323), APC-conjugated anti-mouse F4/80 mAb (123115), PE-conjugated anti-mouse CD19 mAb (152407), PE-Cy7-conjugated anti-mouse CD45.2 (Ly5.2) mAb (109829), APC-conjugated anti-mouse CD44 mAb (103011), APC-Cy7-conjugated anti-mouse CD62L mAb (104427), PE-conjugated anti-mouse CD153 mAb (106405), PerCP/Cy5.5-conjugated anti-mouse CD184 (CXCR4) mAb (146509), PerCP/Cy5.5-conjugated anti-mouse CD185 (CXCR5) mAb (145507), fluorescein isothiocyanate (FITC)–conjugated anti-mouse CD279 (PD-1) mAb (135213), and FITC–conjugated anti-mouse CD326 (EpCAM) mAb (118207), which were obtained from BioLegend (San Diego, CA, USA). A Canto II flow cytometer (BD Biosciences, San Jose, CA, USA) was used to identify cell populations according to surface expression profiles. Flow cytometric data were analyzed using FlowJo software v10 (BD Biosciences). The representative gating strategy for analyzing SA-T cells was reported previously [51]. 

### 4.10. Immunofluorescence Staining

Frozen sections of salivary gland tissue were fixed with methanol/acetone (1:1), blocked using 5% normal goat serum (WAKO)/0.3% Triton X-100 (Sigma) in phosphate-buffered saline (PBS), and stained with FITC-conjugated anti-mouse CD326 (EpCAM) mAb (118207, BioLegend), Alexa Fluor 647-conjugated anti-mouse CD4 mAb (100426, BioLegend), anti-mouse CD153 mAb (14-1531-85, Thermo Fisher Scientific), and an anti-mouse CXCL13 polyclonal antibody (PA1-29046, Thermo Fisher Scientific). Alexa Fluor 555-conjugated anti-rat IgG (H+L) (4417) and Alexa Fluor 594-conjugated anti-rabbit IgG (H+L) (8889), which were obtained from Cell Signaling Technology, were used as the secondary antibody. These antibodies were diluted with the Can Get Signal immunostain solution (Toyobo, Osaka, Japan). After washing 3 times with PBS, nuclear DNA was stained with ProLong Diamond Antifade Mountant with DAPI (Thermo Fisher Scientific). Images were acquired using the KEYENCE BZ-X800 microscope (Osaka, Japan), and processed with the full focus function of the BZ-X800 Analyzer software (Itasca, IL, USA)unless otherwise stated. 

### 4.11. SPiDER-βGal Staining

Frozen sections of salivary gland tissue were fixed in 10% phosphate-buffered formaldehyde (pH 7.2) at RT for 20 min, washed in PBS, and immersed in SPiDER-βGal staining solution (SG03, Dojindo Laboratories, Kumamoto, Japan) at 37 °C for 1 h, according to the manufacturer’s instructions. Imaging was performed after washing with PBS. 

### 4.12. In Vitro Chemotactic Migration Assay

After spleen cells were homogenized and red blood cells hemolyzed, CD4^+^ T cells were collected using a CD4^+^ T cell isolation kit (Miltenyi Biotec, Gladbach, Germany). After serum starvation in 0.1% bovine serum albumin (BSA, Sigma-Aldrich)-RPMI 1640 medium (Sigma-Aldrich) for 24 h, CD4^+^ T cells were plated (3×10^5^ cells in 350 μL) in culture plate inserts (3.0 μm pore size) (Falcon, NY, USA). An equal volume of medium containing CXCL13 (0–4 μg/mL) (BioLegend) was added to the lothermower chamber in 350 μL of 0.1% BSA-RPIM 1640 medium, and cells were then cultured at 37 °C for 6 h. The number of migrated cells was analyzed by flow cytometry.

### 4.13. Statistical Analysis

The significance of differences was evaluated by the Student’s unpaired t-test or Dunnett’s multiple comparison test after an analysis of variance (ANOVA) using GraphPad InStat [57]. Values of *p* < 0.05 were considered to be significant.

## Data Availability

Data that support the results of the present study are available from the corresponding author upon reasonable request.

## Supplementary Materials

The following are available online at https://www.mdpi.com/1422-0067/22/5/2302/s1.
