# Supplementary material for: Chemokines Up-Regulated in Epithelial Cells Control Senescence-Associated T Cell Accumulation in Salivary Glands of Aged and Sjögren’s Syndrome Model Mice
Source: Int J Mol Sci. 2021 Feb 25;22(5):2302. doi: 10.3390/ijms22052302 (PMC7956724; doi:10.3390/ijms22052302)
Supplement: Supplementary file 1 [file ijms-22-02302-s001.pdf]

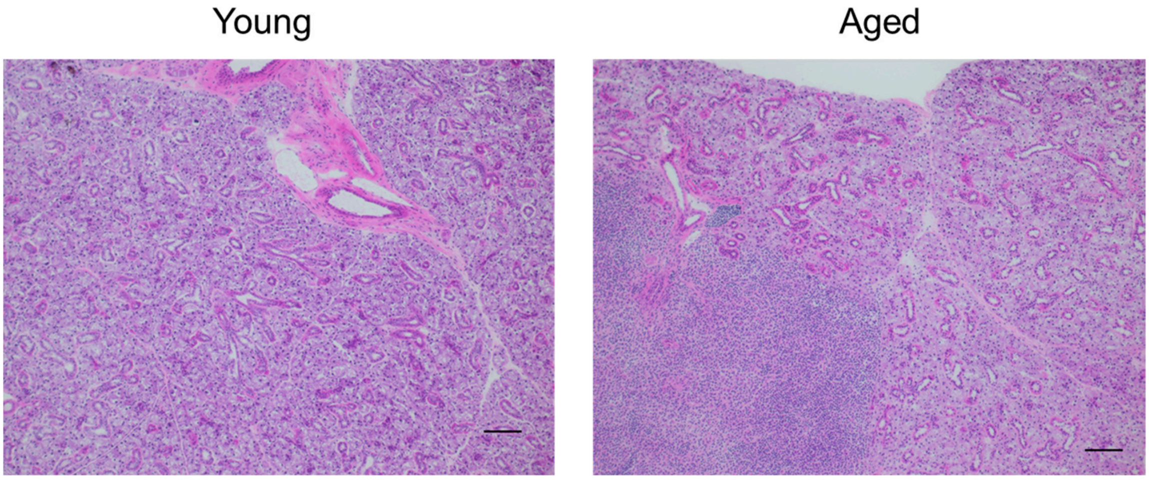

Figure S1. Hematoxylin and eosin staining in salivary glands of female young and aged mice. Bars = 100 μm

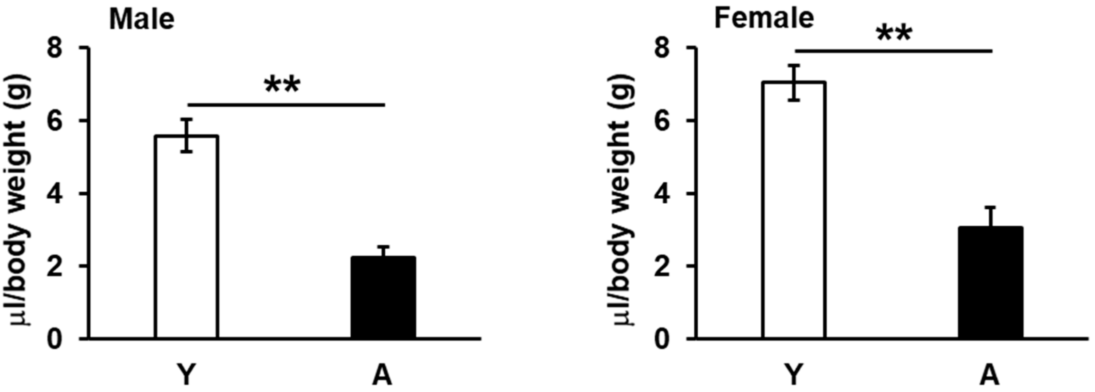

Figure S2. Secretion of saliva after an intraperitoneal injection of pilocarpine in young (Y) or aged (A) mice (male: n = 4 mice per group, female: n = 5 mice per group). Values are shown as means ± SEM. **$p<0.01$ (the Student's unpaired *t*-test).

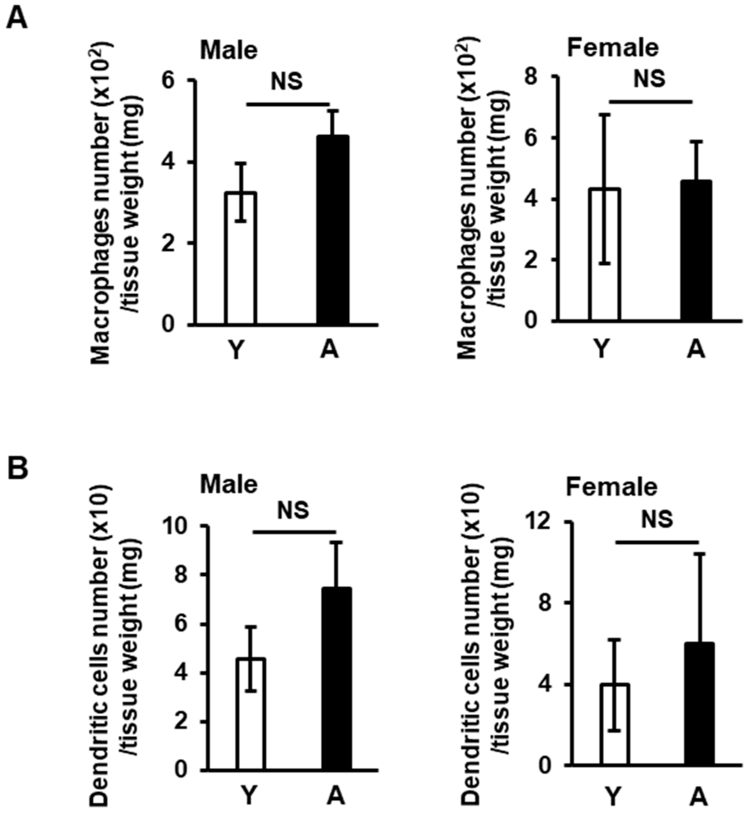

Figure S3. Numbers of $CD11b^{+}$ $F4/80^{+}$ macrophages (**A**) and $CD11b^{+}$ $CD11c^{+}$ dendritic cells (**B**) in salivary glands of young (Y) and aged (A) C57BL/6N mice were determined by flow cytometry. Left and right graph shows male and female mice, respectively. Values are presented as the means ± SEM. (Male: n = 6 mice per group, Female: n = 4-5 mice per group). NS, not significant (Student's unpaired *t*-test).

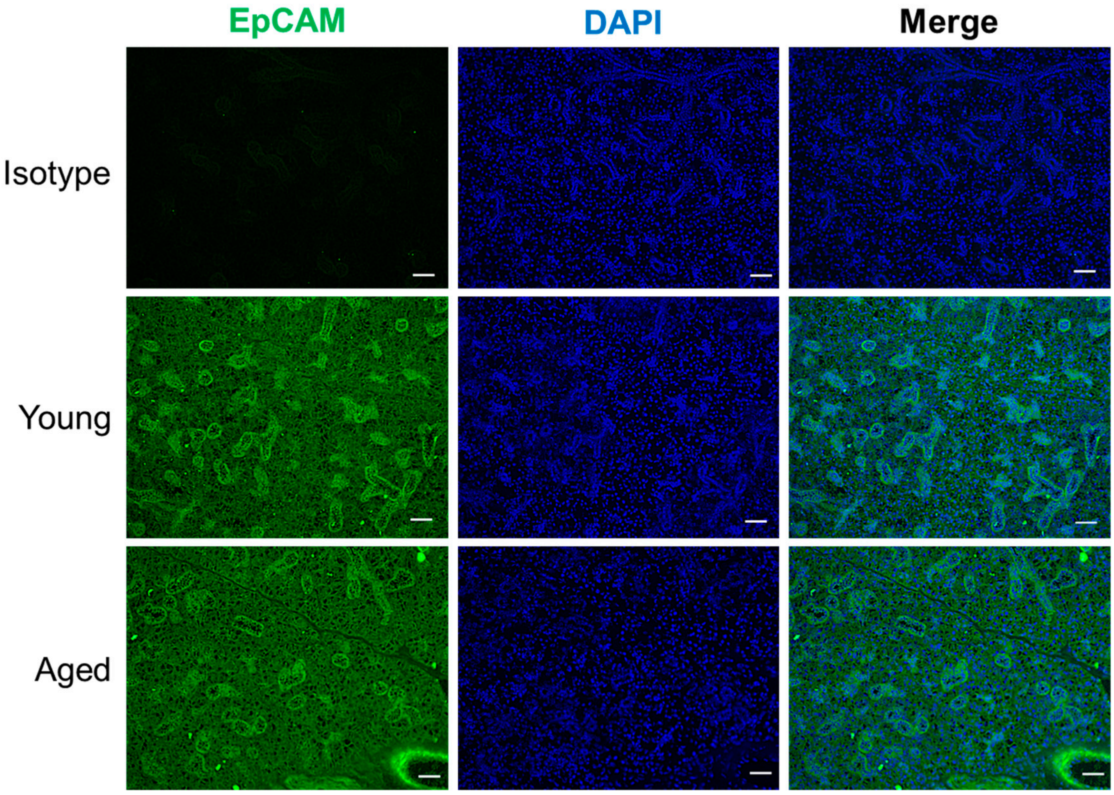

Figure S4. EpCAM expression in the salivary glands of young and aged mice as detected by immunofluorescence analysis using FITC–conjugated anti-mouse EpCAM mAb. Nuclei were stained with DAPI. Bars = 50 μm

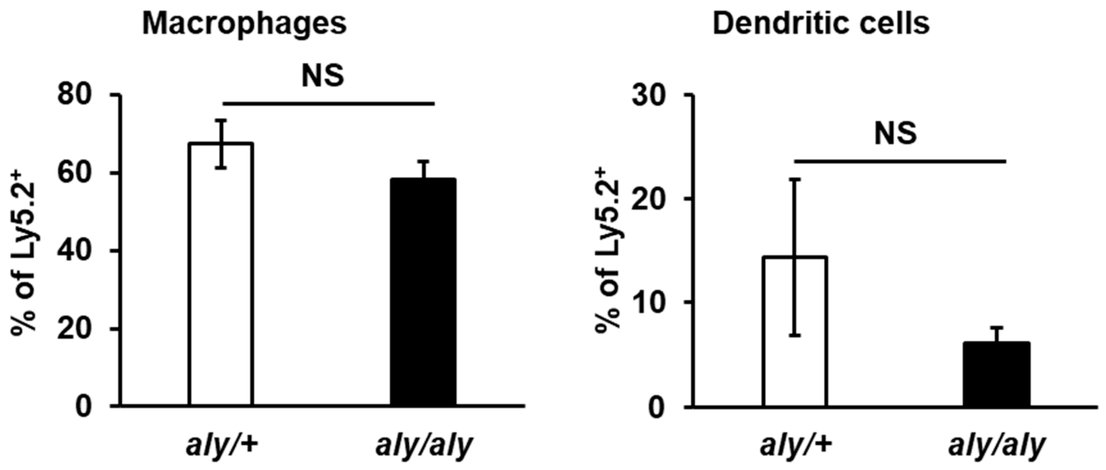

Figure S5. Frequencies of CD11b$^+$ F4/80$^+$ macrophages and CD11b$^+$ CD11c$^+$ dendritic cells gated on Ly5.2$^+$ cells in salivary glands of *aly/+* and *aly/aly* mice were assessed by flow cytometry (n = 3-5 mice per group). Values are shown as means ± SEM. NS, not significant (the Student's unpaired *t*-test).

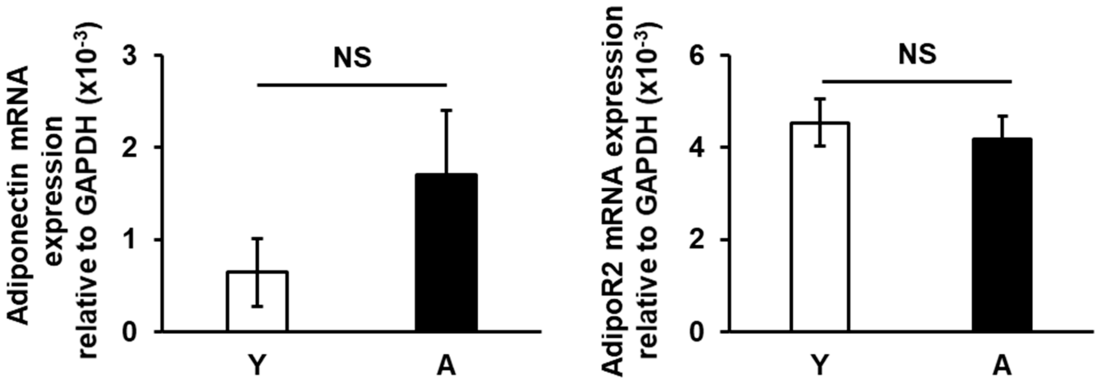

Figure S6. Influence of aging on adiponectin and adipoR2 mRNA expression in salivary glands. Graphs show results in young (Y) and aged (A) mice (N = 7-8). Values are presented as means ± SEM. NS, not significant (the Student's unpaired *t*-test).

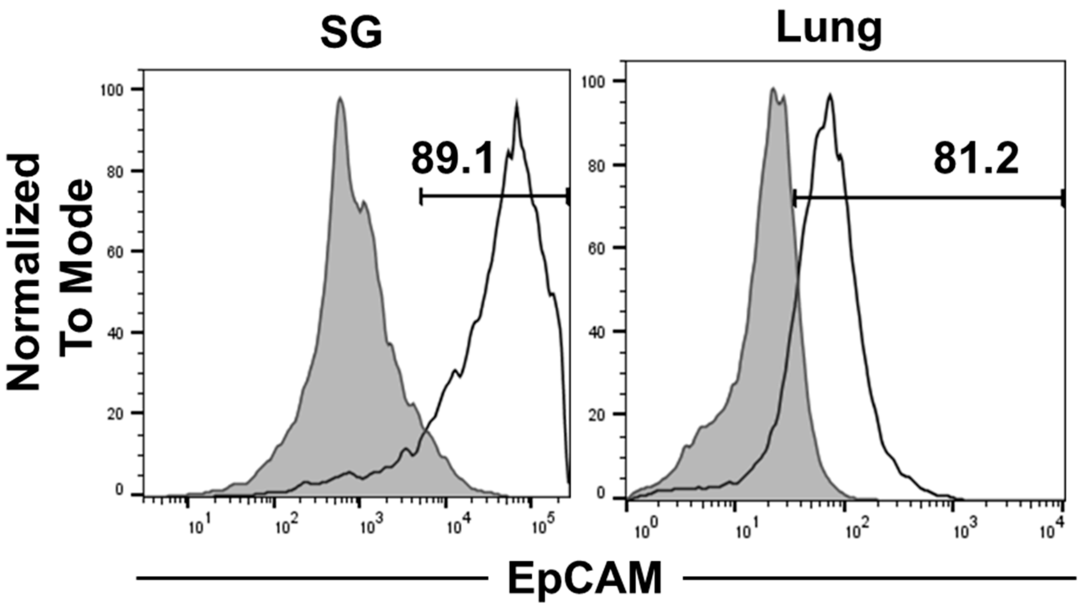

Figure S7. Epithelial cells were isolated using CD326 (EpCAM) Microbeads from murine salivary glands (SG) and lung. Cells were stained with an isotype control or FITC-conjugated CD326 (EpCAM) antibody, and analyzed by flow cytometry. Cell debris was excluded from the analysis based on scatter signals.
